# Immediate sensitivity to structural constraints in pronoun resolution

**DOI:** 10.3389/fpsyg.2014.00630

**Published:** 2014-06-27

**Authors:** Wing-Yee Chow, Shevaun Lewis, Colin Phillips

**Affiliations:** ^1^Department of Linguistics, University of MarylandCollege Park, MD, USA; ^2^Basque Center on Cognition, Brain and LanguageDonostia – San Sebastián, Spain; ^3^Department of Cognitive Science, Johns Hopkins UniversityBaltimore, MD, USA; ^4^Program in Neuroscience and Cognitive Science, University of MarylandCollege Park, MD, USA

**Keywords:** pronoun resolution, Principle B, memory retrieval, self-paced reading, eye-tracking

## Abstract

Real-time interpretation of pronouns is sometimes sensitive to the presence of grammatically-illicit antecedents and sometimes not. This occasional sensitivity has been taken as evidence that structural constraints do not immediately impact the initial antecedent retrieval for pronoun interpretation. We argue that it is important to separate effects that reflect the initial antecedent retrieval process from those that reflect later processes. We present results from five reading comprehension experiments. Both the current results and previous evidence support the hypothesis that agreement features and structural constraints immediately constrain the antecedent retrieval process for pronoun interpretation. Occasional sensitivity to grammatically-illicit antecedents may be due to repair processes triggered when the initial retrieval fails to return a grammatical antecedent.

## Introduction

This paper is concerned with how different kinds of linguistic constraints are used in memory retrieval processes in the course of real-time comprehension. We focus on third-person pronouns for two reasons. First, the interpretation of such pronouns almost always requires the identification of an antecedent from the previous discourse, so they reliably trigger memory retrieval processes in comprehension. Second, the dependency between a pronoun and its antecedent is subject to several kinds of linguistic constraints. Thus, the outcome of the antecedent retrieval process is potentially quite informative about whether the memory system is able to take advantage of different kinds of linguistic constraints to aid sentence processing.

We consider two broad types of constraints on pronominal dependencies. *Agreement* constraints require that the pronoun and its antecedent share certain features, such as number, person, gender, and animacy. For example, in (1), ‘Mary’ cannot be the antecedent for ‘him’ because it mismatches the pronoun in gender. *Structural* constraints require that the antecedent bear certain relations to the pronoun in the syntactic and discourse representations. We focus on the structural constraint known as Binding Principle B (Chomsky, 1981): roughly, a pronoun cannot be bound by an antecedent within its local clause. In (1), ‘Peter’ cannot be the antecedent for ‘him’ because it would bind the pronoun from within the local clause.

(1) Bill explained to Mary that Peter had deceived him.

Note that for the purposes of this paper, it is sufficient to understand Principle B as a descriptive generalization. We are only concerned with which elements are potential antecedents, and which are not. This approach therefore abstracts away from questions about how the distribution and interpretation of pronouns should be explained at the syntactic, semantic, and pragmatic level (Reinhart, 1983; Grodzinsky and Reinhart, 1993). We are also restricting ourselves to pronouns with intrasentential antecedents, and thus will not be considering the role of various discourse-level structural constraints on extrasentential antecedents.

The combination of agreement and structural constraints substantially narrows the field of potential antecedents for a pronoun. In (1), for example, three entities are mentioned before ‘him,’ but only ‘Bill’ is a possible antecedent. Thus, it seems that an efficient comprehension system would take advantage of all available constraints as soon as possible—in the initial retrieval of an antecedent. However, antecedent retrieval relies on memory processes, and it is by no means guaranteed that the human memory system is capable of using any and all linguistic constraints to restrict retrieval. It is therefore an open empirical question which kinds of constraints are used immediately in the initial retrieval, and which have their effect later, as “filters” on the results of the retrieval.

Previous research has demonstrated that comprehenders are sensitive to agreement constraints very early in the process of pronoun interpretation. They rapidly and accurately identify feature-matching entities in the discourse (Arnold et al., 2000) and detect feature mismatches between a pronoun and its supposed antecedent (Osterhout and Mobley, 1995; Carreiras et al., 1996; Van Berkum et al., 2004). Based on these findings, we consider it uncontroversial that the initial antecedent retrieval process takes advantage of agreement features to restrict the set of candidates.

On the other hand, while many previous studies have examined the role of Principle B in real-time pronoun interpretation, their results have led to divergent conclusions (see Nicol and Swinney, 2003 and Sturt, 2013 for reviews). While existing accounts differ on many aspects, one critical point of contention among them concerns whether and how structural constraints impact the initial antecedent-retrieval processes. Some have argued that only structurally acceptable potential antecedents are considered during the early stages of processing (e.g., Nicol and Swinney, 1989; Clifton et al., 1997, 1999; Lee and Williams, 2008; Patterson et al., 2014), while others have contended that structurally unacceptable candidates can be retrieved initially if they match the pronoun in features (e.g., Badecker and Straub, 2002; Kennison, 2003; Runner et al., 2006). We hope to clarify the kind of evidence that would be necessary to support each of these alternatives by distinguishing different forms of sensitivity to structurally unacceptable potential antecedents. We argue that the apparently conflicting results from previous studies, as well as the results from our own studies, are all consistent with the simultaneous use of agreement and structural constraints in the initial antecedent retrieval process.

We abstract away from other differences among existing accounts and consider two competing hypotheses that differ minimally. The *Agreement First* hypothesis is that the initial retrieval process uses agreement features, but not Principle B, to restrict the set of potential antecedents for a pronoun. Under the *Simultaneous Constraints* hypothesis, the initial retrieval process uses both agreement and structural constraints simultaneously. Here we adopt the operational definition that, when the retrieval process “uses” or “implements” a constraint, the initial set of candidate antecedents does not contain any elements that would be ruled out by that constraint. The retrieval either returns all elements allowed by the constraint, or returns any one of those elements with equal probability. Let's return to our example in (1). Under the Agreement First hypothesis, the initial set of candidate antecedents would contain both ‘Bill’ and ‘Peter’ (or either one with some probability), since both match ‘him’ in agreement features. The structurally unacceptable ‘Peter’ would have to be ruled out later. Under the Simultaneous Constraints hypothesis, the initial candidate set would contain only ‘Bill.’

This particular comparison reflects a slight change of perspective since the earlier studies on pronouns and the hypotheses considered here do not map directly onto any existing account. In particular, while earlier proposals asked whether structural constraints could preempt agreement features as an initial filter on the set of candidate antecedents (e.g., Nicol and Swinney, 1989; Clifton et al., 1997), we now take for granted that agreement features are used in the earliest stages of pronoun processing. The early sensitivity to agreement features fits naturally with models of sentence processing that incorporate cue-based retrieval in a content-addressable memory system (e.g., Lewis and Vasishth, 2005; Martin and McElree, 2008). What remains to be determined is when and how structural constraints play their role.

The Simultaneous Constraints hypothesis should be distinguished from previous accounts involving multiple weighted constraints, in which different constraints can be weighted and applied probabilistically (e.g., Badecker and Straub, 2002; Runner et al., 2006). For example, under Badecker and Straub's (2002) “interactive parallel constraints” hypothesis, a structural constraint can be outweighed by a discourse prominence constraint. As such, even evidence for the retrieval of a structurally unacceptable potential antecedent can be fully compatible with the immediate application of a structural constraint. In contrast, in the current formulation the simultaneously applied constraints are deterministic, so that the retrieval process cannot return an element that is ruled out by any one of them. Thus, we can falsify this hypothesis when we obtain evidence that an element that is ruled out by one (or more) of the constraints is retrieved initially.

To examine the effects of structural constraints on real-time pronoun interpretation, reading studies have generally used feature-mismatch paradigms (e.g., Clifton et al., 1999; Badecker and Straub, 2002; Lee and Williams, 2008). For example, the paradigm illustrated in (2) orthogonally manipulates the gender match between the pronoun and two potential antecedents: the structurally acceptable main clause subject (‘John’/‘Jane’), and the structurally unacceptable embedded clause subject (‘Bill’/‘Mary’). Reading times at and following the pronoun are considered indicative of the relative difficulty of resolving the reference of the pronoun (e.g., Carreiras et al., 1996; Clifton et al., 1997).

(2) a. John thought that Bill liked him a lot.
  b. John thought that Mary liked him a lot.
  c. Jane thought that Bill liked him a lot.
  d. Jane thought that Mary liked him a lot.

In (2), the main clause subject is the only structurally acceptable potential antecedent for the pronoun ‘him.’ When this subject mismatches the pronoun in gender, as in (c) and (d), the sentences are considered ungrammatical. (Although an antecedent outside the sentence is technically possible, such sentences are initially perceived as ungrammatical when no context is provided. Presumably it takes some time to accommodate the lack of antecedent by inventing a potential context with an appropriate antecedent). Thus, sensitivity to the features of the structurally acceptable candidate in reading times at or following the pronoun is termed a *grammaticality effect*. On the other hand, since Principle B rules out the embedded clause subject as a potential antecedent for the pronoun, its features are irrelevant to the acceptability of the sentence. Thus (a) and (b) are equally acceptable, and (c) and (d) are equally unacceptable. Any sensitivity to the features of structurally unacceptable potential antecedents is broadly termed an *interference effect*.

Our predictions for each hypothesis depend on the assumption that some cost will be incurred if the context does not contain a potential antecedent that satisfies the constraints on the initial antecedent retrieval process. This assumption is compatible with most popular conceptions of memory retrieval mechanisms. On the one hand, if retrieval is deterministic and exhaustive, returning all and only the candidates that satisfy all the constraints, then a lack of satisfactory potential antecedents in the context will result in retrieval failure. Since a pronoun cannot be interpreted without identifying its antecedent, retrieval failure would trigger an error signal or a repair or reanalysis process, either of which would be observable as increased reading times. On the other hand, if retrieval is probabilistic, returning a single candidate with greater or lesser likelihood depending on how fully it satisfies the constraints, then a lack of satisfactory potential antecedents in the context will result in the retrieval of partial matches. In this case, the additional cost arises from the need to rule out partial matches after they have been retrieved. Note that this “filter” would be separate from and prior to a filter based on constraints that were not active in the initial retrieval process.

Let us now consider the predictions of each of our alternative hypotheses. Under the Simultaneous Constraints hypothesis, agreement and structural constraints are both applied during the initial retrieval process, resulting in a set of feature-matching and structurally acceptable candidate antecedents. In (c) and (d), where the structurally acceptable noun phrase mismatches the pronoun in gender, the retrieval process will either—depending on one's preferred model of memory retrieval—fail to return any candidates, or return partial matches that must be ruled out. Either option would be costly. The Simultaneous Constraints hypothesis would therefore predict a grammaticality effect: longer reading times after the pronoun in (c) and (d) compared to (a) and (b). In fact, previous studies have consistently reported grammaticality effects (e.g., Clifton et al., 1999; Badecker and Straub, 2002; Lee and Williams, 2008).

Under the Agreement First hypothesis, structural constraints are not applied in initial antecedent retrieval, resulting in a set of feature-matching candidates that may or may not be structurally acceptable. In (2), (a) to (c) all contain at least one male name, matching the pronoun ‘him’ in features. The retrieval should only encounter difficulties in (d), which contains no feature-matching names. Thus, reading times after the pronoun in (a) to (c) should pattern together, contrasting with longer reading times in (d). Since reading times would differ between (c) and (d) based solely on the features of a structurally unacceptable potential antecedent, this is a type of interference effect. We will refer to this pattern as a *facilitative interference effect*, since the presence of a feature-matching but structurally unacceptable potential antecedent reduces reading times relative to (d).

Only a facilitative interference effect constitutes sufficient evidence to support the Agreement First hypothesis and rule out the Simultaneous Constraints hypothesis. It is therefore essential to emphasize that a facilitative interference effect has never been reported for pronouns. No existing evidence rules out the possibility that structural constraints restrict the initial antecedent retrieval process.

However, previous studies have occasionally reported *inhibitory interference effects*. These are of two types, distinguished by whether they arise in grammatical or ungrammatical sentences. Badecker and Straub (2002) observed a *multiple match effect*: when the structurally acceptable candidate matched the pronoun in gender (i.e., in grammatical sentences), reading times were longer when the structurally unacceptable candidate also matched, as in (2a), compared to when it did not (2b). Badecker and Straub interpreted this result as an effect of competition between the two feature-matching candidates. Crucially, however, since they also observed a grammaticality effect, not a facilitative interference effect, the results cannot be taken as evidence for the Agreement First hypothesis. At most, this pattern might support weakening the Simultaneous Constraints hypothesis, so that structural constraints interact with agreement constraints probabilistically, rather than deterministically restricting the initial set of candidate antecedents. To our knowledge, Badecker and Straub (2002) are the only authors to report a multiple match effect in a reading study. It is also worth noting that Clackson et al. (2011) found a similar effect in a visual world eye-tracking study.

A second type of inhibitory interference effect has also been reported. Kennison (2003) examined reading times in sentences like (3) where no structurally acceptable antecedent was available within the sentence. Reading times were longer when a structurally unacceptable potential antecedent matched the pronoun in features (‘Carl’), compared to when it did not (‘Susan’). We will call this type of pattern an *ungrammatical match effect*. Based on this finding, Kennison argued that structurally unacceptable potential antecedents are included in the initial set of candidate antecedents. Their presence delays the point when the comprehender can terminate the search for an antecedent and assume that the intended antecedent is an unmentioned discourse entity.

(3) {Carl/Susan} watched him yesterday during the open rehearsals of the school play.

Sturt (2003) observed a similar ungrammatical match effect in late processing measures in an eye-tracking study on reflexives. However, this study, unlike Kennison's, included a manipulation of the gender match of a structurally acceptable potential antecedent as well as the unacceptable one. There were early grammaticality effects: first-fixation and first-pass reading times on the reflexive were faster in sentences like (4), where the structurally acceptable potential antecedent (‘the surgeon’) matched the reflexive in stereotypical gender, compared to sentences like (5) where it mismatched. The ungrammatical match effect emerged later: in sentences like (5), second pass reading times on the pronoun were longer when the structurally unacceptable potential antecedent matched the reflexive in features (‘She’) than when it did not (‘He’). Based on the combination of early grammaticality effects and late inhibitory interference, Sturt proposed that the initial set of candidate antecedents is structurally constrained (by Principle A, in the case of reflexives), but structurally unacceptable potential antecedents may be considered at a later stage if no acceptable candidates are retrieved initially.

(4) … {She/He} remembered that the surgeon had pricked himself with a used syringe needle. …
(5) … {She/He} remembered that the surgeon had pricked herself with a used syringe needle. …

In summary, different forms of sensitivity to structurally acceptable potential antecedents warrant distinct interpretations (cf. Sturt, 2013). Facilitative interference provides the only clear evidence for the Agreement First hypothesis. Other forms of interference are consistent with the Simultaneous Constraints hypothesis: they may reflect other properties of the processing system or later stages of processing. Thus, given our stricter interpretation of interference effects, the previous literature provides no positive evidence for the Agreement First hypothesis and is consistent with the Simultaneous Constraints hypothesis.

We had two goals for our experiments: to probe further for facilitative interference, and to investigate the causes of the other attested forms of interference. In Experiment 1 we show that comprehenders are immediately sensitive to the structural constraints on pronoun interpretation, regardless of the similarity between the candidate antecedents and their linear distance from the pronoun. We found robust effects of grammaticality, but no interference effects of any kind. In Experiment 2 we attempted to reconcile the discrepancy between our results and Badecker and Straub's (2002) findings in Experiment 2 by directly reproducing their experiment. We replicated our findings from Experiment 1, observing a clear effect of grammaticality but no interference effects. In three additional experiments, we never observed a multiple match effect. Thus, our results support the stronger version of the Simultaneous Constraints hypothesis: structural constraints immediately restrict the initial antecedent retrieval process.

## Experiment 1

Experiment 1 had two goals. First, we wanted to investigate whether structural constraints immediately restrict the set of candidate antecedents for a pronoun. Second, we wanted to explore the possibility that superficial differences in experimental materials may have caused the discrepancies among previous findings.

The first goal of Experiment 1 was to examine whether structural constraints immediately restrict the set of candidate antecedents. We used the feature mismatch paradigm, manipulating the gender match between the object pronoun ‘him’ and two candidate antecedents: the structurally acceptable main clause subject and the structurally unacceptable embedded clause subject. According to the Simultaneous Constraints hypothesis, the initial retrieval process only returns candidate antecedents that satisfy both feature-match and structural constraints. We should observe only a main effect of grammaticality—gender match of the structurally acceptable candidate—in reading times at the pronoun and subsequent words. According to the Agreement First hypothesis, the initial retrieval process relies on feature matching alone and structural constraints only impact later stages of processing. Under this hypothesis, we should observe an interaction between the two factors in a pattern of facilitative interference. Specifically, reading times should be longer when neither potential antecedent matches the pronoun in gender, compared to the other three conditions where at least one of the potential antecedents matches the pronoun in features. The Agreement First hypothesis would also be consistent with a concurrent or subsequent multiple match effect, if retrieving multiple feature-matching candidates leads to competition-related costs.

The second goal of Experiment 1 was to explore the possibility that superficial differences in experimental materials may have caused the discrepancies among previous findings. We focused on two properties of the materials: (1) similarity between the structurally acceptable and unacceptable candidate antecedents; and (2) linear distance between the pronoun and the structurally acceptable candidate. Even if structural constraints can immediately restrict the set of candidate antecedents during the initial retrieval process (*Simultaneous Constraints* hypothesis), similarity-based interference (e.g., Gordon et al., 2001) and memory decay (Keppel and Underwood, 1962) may make it more difficult for comprehenders to distinguish between structurally acceptable and unacceptable potential antecedents during retrieval. As a result, when the potential antecedents are more similar or when the distance between the pronoun and the structurally acceptable potential antecedent is greater, comprehenders may be more likely to retrieve a feature-matching but structurally unacceptable candidate from a noisy memory representation and show facilitative interference. To explore the effects of these factors, we manipulated the properties of the embedded subject (the structurally unacceptable potential antecedent), as illustrated in Table 1.

**Table 1:** **Experimental conditions and sample materials in Experiment 1**.

	**Embedded match**	**Embedded mismatch**
**COMMON NOUN**		
Main clause match (grammatical)	Ethan discovered that the analyst had mocked him mercilessly for singing karaoke after drinking…	Ethan discovered that the receptionist had mocked him mercilessly for singing karaoke after drinking…
Main clause mismatch (ungrammatical)	Paige discovered that the analyst had mocked him mercilessly for singing karaoke after drinking…	Paige discovered that the receptionist had mocked him mercilessly for singing karaoke after drinking…
**MODIFIED COMMON NOUN**		
Main clause match (grammatical)	Ethan discovered that the analyst who attended the office party had mocked him mercilessly for singing karaoke after drinking…	Ethan discovered that the receptionist who attended the office party had mocked him mercilessly for singing karaoke after drinking…
Main clause mismatch (ungrammatical)	Paige discovered that the analyst who attended the office party had mocked him mercilessly for singing karaoke after drinking…	Paige discovered that the receptionist who attended the office party had mocked him mercilessly for singing karaoke after drinking…
**PROPER NAME**		
Main clause match (grammatical)	Ethan discovered that Ronald had mocked him mercilessly for singing karaoke after drinking…	Ethan discovered that Marissa had mocked him mercilessly for singing karaoke after drinking…
Main clause mismatch (ungrammatical)	Paige discovered that Ronald had mocked him mercilessly for singing karaoke after drinking…	Paige discovered that Marissa had mocked him mercilessly for singing karaoke after drinking…

Previous studies vary in the similarity between the potential antecedents in the sentence. For example, Badecker and Straub (2002) used sentences where both potential antecedents were proper names, and observed a multiple match effect. By contrast, Lee and Williams (2008) used a common noun as the structurally acceptable candidate and a proper name as the unacceptable candidate, and did not observe any interference effects. In our experiment, the main clause subject was always an unambiguously gendered proper name (e.g., ‘Ethan’ or ‘Paige’). We manipulated the similarity between the structurally acceptable and unacceptable candidates by using either another unambiguously gendered proper name (e.g., ‘Ronald,’ ‘Marissa’) or a gender-biased common noun (e.g., ‘the analyst,’ ‘the receptionist’) as the embedded subject, as shown in Table 1.

Previous studies also vary in the distance between the pronoun and potential antecedents. A previous eye-tracking study by Ehrlich and Rayner (1983) found that reading times following a pronoun are longer when its antecedent is further away (cf. Walker et al., 1983). In our experiment, we increased the linear distance between the pronoun and the structurally acceptable candidate by modifying the common noun embedded subject with a subject relative clause or a prepositional phrase (e.g., ‘the analyst who attended the office party’), as shown in Table 1.

### Methods

#### Participants

Thirty-six students (26 female, mean age = 20 years, range between 18 and 28) from the University of Maryland, College Park participated in this experiment. All participants were native speakers of English and had normal or corrected-to-normal vision. All participants gave informed consent and received course credit for their participation. Procedures for this experiment as well as Experiments 2–5 were approved by the Internal Review Board of the University of Maryland, College Park.

#### Design and materials

We crossed two levels of *main clause subject gender* (match/mismatch) and *embedded subject gender* (match/mismatch) with three levels of *embedded subject type* (proper name/common noun/modified common noun) to result in a 2 × 2 × 3 within-participant design. The pronoun was always ‘him,’ since the feminine pronoun ‘her’ is ambiguous between an object pronoun and a possessive pronoun. We created 60 sets of experimental sentences. Each set included twelve variants, one in each condition. A sample set is shown in Table 1. A complete set of experimental stimuli are available in the Supplementary Materials.

The main clause subject was always an unambiguously gendered proper name. The embedded subject was either an unambiguously gendered proper name (e.g., ‘Ronald,’ ‘Marissa’), a gender-biased common noun (e.g., ‘the analyst,’ ‘the receptionist’), or a gender-biased common noun modified with a subject relative clause or a prepositional phrase (e.g., ‘the analyst who attended the office party’).

The gender-biased nouns were selected based on norming data from Kennison and Trofe (2003) and the intuitions of a native speaker. We collected gender bias ratings for all gender-biased nouns used in this experiment and Experiments 3–5 using Amazon Mechanical Turk. Twenty participants (9 female, mean age = 25 years, range between 21 and 28) rated each noun on a scale from 1 (most likely female) to 7 (most likely male). Overall the results support our choice of nouns. In Experiment 1, the female-biased nouns had an average rating of 2.5 (all of which had an average rating below 4) and the male-biased nouns had an average rating of 5.3 (57 out of 60 had an average rating above 4). The median rating difference between the female-biased and male-biased nouns within the same item was 2.6; 58 of the 60 pairs had mean differences of at least 1.

The 60 item sets were divided into 12 lists, such that each list contained exactly one version of each item and 5 items in each condition. Each list also contained 60 filler sentences, which varied in length and syntactic complexity and contained other referential expressions (e.g., proper names and gender-neutral nouns) and anaphors (e.g., feminine pronouns and reflexives). A third of the experimental and filler sentences were followed by a yes/no comprehension question to ensure that participants were attending to the stimuli. The comprehension questions never referred to the referential dependency between the pronoun and its antecedent. The order of experimental and filler sentences was randomized across participants.

#### Procedure

The experiment was conducted in a quiet room on a desktop PC. Participants read the sentences in a word-by-word, self-paced moving window task (Just et al., 1982) implemented with the Linger software package (Rohde, 2003). Each trial began with the sentence masked by underscores (___), with the words separated by spaces. Participants began a trial by pressing the spacebar, upon which the first word of the sentence appeared. They continued to press the spacebar to read each successive word. As each word appeared, the previous word was remasked. Participants were instructed to read at a natural pace and to make sure they understood what they were reading so that they could respond to comprehension questions accurately. Reaction times (RTs) were measured for each word from the time it appeared on the screen until the spacebar was pressed for the next word. In a third of the items, a comprehension question appeared after the last word in the sentence was read. Participants responded by pressing the “F” key for “Yes” and the “J” key for “No,” and could then proceed to the next trial by pressing the spacebar. The experimental session was preceded by 6 practice trials to familiarize the participant with the procedure. Testing sessions lasted approximately 35 minutes.

#### Analysis

Details of data analysis were consistent across all self-paced reading experiments (Experiments 1–4) and are presented for Experiment 1 only. In each experiment, only data from participants with at least 75% accuracy on the comprehension questions (and on the probe identification task in Experiment 2) were used in the analyses. No participants were excluded due to poor accuracy in Experiment 1. Trials containing RTs greater than 2000 ms were excluded from the analysis. This affected 3.4% of the data for Experiment 1.

Average reading times were compared across conditions in the following regions of interest: the pronoun itself (*pronoun*) and the two words immediately following the pronoun (*pronoun+1* and *pronoun+2*). Data for each of the regions of interest were entered into a 2 × 2 × 3 repeated measures ANOVA with *main clause match, embedded match*, and *embedded subject type* as within-participant and within-item factors. ANOVAs were computed on the participant means collapsing over items (F1), and on the item means collapsing over participants (F2). Below we report comparisons that revealed a statistically significant difference in at least one of the by-participant and by-item analyses. Since the manipulation of embedded subject type resulted in superficial differences in the materials (e.g., sentence length), effects of embedded subject type are not interpretable unless they interact with the effects of main clause match and/or embedded match. Therefore, only effects involving main clause match or embedded match are discussed. Further, a 2 × 2 repeated measures ANOVA with *main clause match* and *embedded match* were conducted on each level of *embedded subject type* when it interacted with one or both of the other factors.

### Results

Participants answered the comprehension questions with an average of 87.8% accuracy.

**Figure 1:**
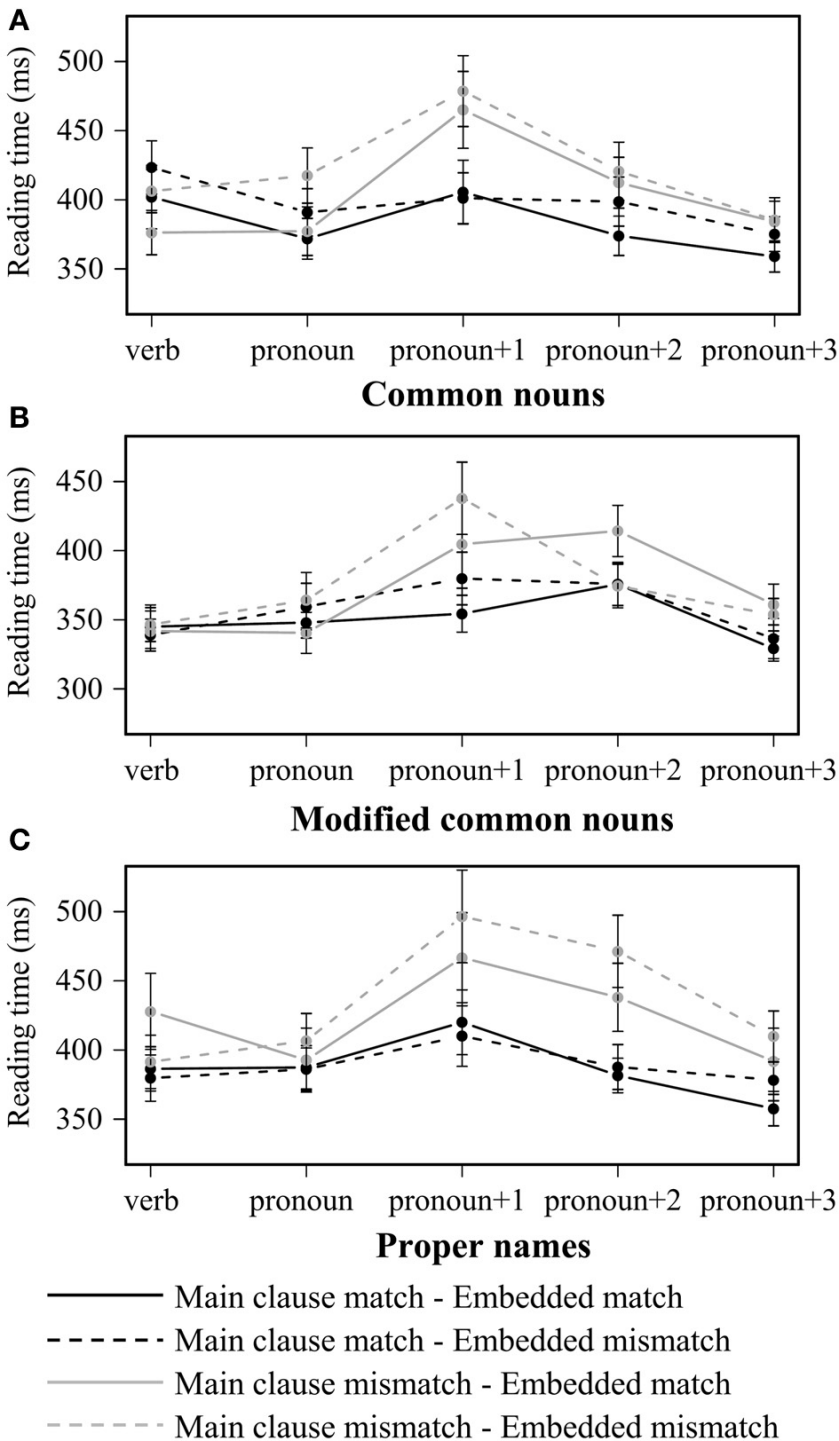
**Word-by-word reading times for (A) the common noun, (B) modified common noun and (C) proper name conditions in Experiment 1**. Error bars indicate standard error of the participant mean.

Table 2 shows average reading times and standard errors in each region of interest (ROI) across all conditions. Figure 1 shows average reading times starting from the word preceding the pronoun (the embedded verb) to one word following *pronoun+2* across conditions in each level of embedded subject type. The three-way repeated measures ANOVA in the *pronoun* region revealed a significant main effect of *embedded match* in the by-participant analysis [*F*_1(1, 35)_ = 5.84, *p* < 0.05; *F*_2(1, 59)_ = 3.33, *p* = 0.07]: reading times were longer when the embedded subject mismatched the pronoun in gender. A significant main effect of *main clause match* was observed in both the *pronoun+1* [*F*_1(1, 35)_ = 16.12, *p* < 0.001; *F*_2(1, 59)_ = 30.57, *p* < 0.001] and *pronoun+2* [*F*_1(1, 35)_ = 12.24, *p* < 0.01; *F*_2(1, 59)_ = 21.35, *p* < 0.001] regions: reading times were significantly longer when the main clause subject mismatched the pronoun in gender (i.e., a grammaticality effect). This main effect was accompanied by a significant interaction between main clause match and embedded subject type in the *pronoun+2* region [*F*_1(1, 35)_ = 16.49, *p* < 0.001; *F*_2(2, 118)_ = 3.57, *p* < 0.05].

**Table 2:** **Grand average reading times (with standard deviations) in each ROI across all conditions in Experiment 1**.

	**Pronoun**	**Pronoun+1**	**Pronoun+2**
**COMMON NOUN**			
Main clause match, embedded match	372 (15)	406 (23)	374 (14)
Main clause match, embedded mismatch	391 (17)	401 (18)	399 (18)
Main clause mismatch, embedded match	377 (18)	465 (28)	412 (18)
Main clause mismatch, embedded mismatch	418 (20)	479 (26)	421 (21)
**MODIFIED COMMON NOUN**			
Main clause match, embedded match	348 (11)	354 (13)	376 (15)
Main clause match, embedded mismatch	359 (17)	380 (19)	376 (16)
Main clause mismatch, embedded match	341 (15)	405 (32)	414 (19)
Main clause mismatch, embedded mismatch	364 (20)	438 (26)	374 (16)
**PROPER NAME**			
Main clause match, embedded match	387 (16)	420 (23)	382 (13)
Main clause match, embedded mismatch	386 (15)	410 (22)	388 (16)
Main clause mismatch, embedded match	393 (23)	467 (33)	438 (25)
Main clause mismatch, embedded mismatch	407 (20)	497 (34)	471 (26)

To better understand the interaction involving embedded subject type, a two-way repeated measures ANOVA with *main clause match* and *embedded match* was conducted on each level of *embedded subject type* in the *pronoun+2* region. When the embedded subject was a common noun (e.g., ‘the analyst’; Figure 1A), there was a significant main effect of *main clause match* in the by-item analysis [*F*_1(1, 35)_ = 2.60, *p* > 0.1; *F*_2(1, 59)_ = 4.08, *p* < 0.05]. When the embedded subject was a modified common noun (e.g., ‘the analyst who attended the office party’; Figure 1B), the main effect of *main clause match* was not significant (*p* > 0.1), but there was an interaction between *main clause match* and *embedded match* that is significant in the by-participant analysis [*F*_1(1, 35)_ = 5.59, *p* < 0.05; *F*_2(1, 59)_ = 1.91, *p* > 0.1]: gender mismatch between the pronoun and the main clause subject led to longer reading times in the *pronoun+2* region only when the embedded subject matched the pronoun (i.e., an ungrammatical match effect). When the embedded subject was a proper name (e.g., ‘Ronald’; Figure 1C), there was a significant main effect of *main clause match* [*F*_1(1, 35)_ = 14.83, *p* < 0.001; *F*_2(1, 59)_ = 13.59, *p* < 0.001]: reading times were significantly longer when there was a gender mismatch between the pronoun and the main clause subject (i.e., a grammaticality effect).

### Discussion

Experiment 1 had two main findings. First, we observed a robust grammaticality effect: reading times after the pronoun were significantly longer when the only structurally acceptable potential antecedent mismatched the pronoun in gender (a main effect of *main clause match* in both post-pronoun regions). This grammaticality effect was modulated by embedded subject type in the *pronoun+2* region: the effect of grammaticality was largest when both the main clause and embedded subjects were proper names.

Second, we never observed a facilitative interference effect. Overall three-way ANOVAs did not reveal a significant *main clause match* × *embedded match* interaction in any of the ROIs. Although a significant interaction was observed in the two-way ANOVA in the *pronoun+2* region in the modified common noun condition, it showed the opposite pattern: the presence of a feature-matching structurally unacceptable potential antecedent led to longer, rather than shorter, reading times in the absence of an acceptable antecedent. Therefore, neither similarity between the structurally acceptable and unacceptable candidates nor increased distance between the pronoun and the acceptable antecedent resulted in more retrievals of structurally unacceptable potential antecedents. Taken together, the robust sensitivity to the gender of a structurally acceptable potential antecedent and the absence of facilitative inference effects support the Simultaneous Constraints hypothesis. Structural criteria can immediately restrict the set of candidate antecedents during the initial memory retrieval processes.

An unexpected finding of this experiment is the observation of a significant main effect of *embedded match* in the *pronoun* region in the by-participant analysis. This effect did not interact with *embedded subject type* and was not predicted by either hypothesis. A closer inspection of the data suggests that it was mainly carried by the difference in the common noun condition (embedded mismatch: 404 ms vs. embedded match: 374 ms; Figure 1A), which showed a similar difference in reading times in the preceding region (415 ms vs. 389 ms). Therefore, this effect may be spurious and unrelated to pronoun processing.

Although the manipulation of *embedded subject type* never resulted in any facilitative interference effects, it did lead to an interesting interaction between *main clause match* and *embedded subject type* in the *pronoun+2* region. In particular, while main clause mismatch led to significantly longer reading times across all embedded subject types in the *pronoun+1* region, this effect continued to be observed in the *pronoun+2* region only in the proper name condition. That is, when the structurally acceptable and unacceptable potential antecedents were more similar to each other (both were proper names), the grammaticality effect lasted longer. This unexpected pattern could reflect either a more sustained processing disruption or greater variability in the onset time of the disruption.

When the linear distance between the pronoun and the structurally acceptable potential antecedent was lengthened (in the modified common noun condition), we observed a late-emerging ungrammatical match effect. Following the main effect of *main clause match* in the *pronoun+1* region, main clause mismatch led to longer reading times in the *pronoun+2* region only when the embedded subject matched the pronoun. Following Sturt's (2003) proposal for the processing of reflexives, we propose that this inhibitory interference reflects a repair process triggered by an initial failure to retrieve a feature-matching and structurally acceptable antecedent for the pronoun. We take the increased reading times in the *main clause mismatch, embedded match* condition to suggest that a feature-matching antecedent from a structurally unacceptable position may be retrieved when the initial retrieval fails. The observation of an ungrammatical match effect in the modified common noun conditions, but not in the common noun and proper name conditions, suggests that an initial retrieval failure may be more likely to trigger a repair process when the memory representation of the structurally acceptable potential antecedent is less activated due to decay over time. Note, however, that the embedded subject NP was heavier (and more complex) in the modified common noun condition than in the other two conditions. Since a heavier NP may require a more detailed memory representation, the heaviness (or complexity) of the structurally unacceptable potential antecedent may also impact the likelihood of triggering a repair process. More research will be needed to explore the effects of the heaviness of an NP on memory representation and how it might impact memory encoding and retrieval more generally.

Finally, we never observed a multiple match effect: reading times were never longer when both subjects matched the pronoun compared to when only the main clause subject matched the pronoun. As shown in Figure 1, reading times in the multiple match conditions were short in every region, across all three levels of embedded subject type. We aimed to resolve this discrepancy between the current results and Badecker and Straub's (2002) findings in Experiment 2.

## Experiment 2

In Experiment 1 we never observed any sensitivity to the presence of multiple feature-matching candidate antecedents. This contrasts with Badecker and Straub's (2002) repeated observations of a multiple match effect—longer reading times when both candidate antecedents matched the pronoun. We reasoned that, even though the proper name condition in Experiment 1 mirrored Badecker and Straub's (2002; hereafter B&S) Experiment 1, other differences between the experimental materials and procedures might have given rise to the discrepancy in the results. Thus, in Experiment 2, we attempted to directly replicate B&S's Experiment 1, using identical experimental materials and procedures.

We identified three main differences between the materials and procedures used in our Experiment 1 and B&S's Experiment 1. First, while we only used the masculine object pronoun ‘him,’ in order to avoid the ambiguity of the pronoun, B&S used the ambiguous feminine object pronoun ‘her’ in half of the sentences, and analyzed the results from sentences with feminine and masculine object pronoun together. Second, while our participants answered yes/no comprehension questions after a third of the items, B&S's participants performed a probe recognition task after each sentence and answered a yes/no comprehension only after a quarter of the sentences. Furthermore, B&S's participants received auditory feedback on their accuracy for both secondary tasks. Finally, while we presented sentences in a moving-window paradigm, B&S presented each word serially in the center of the screen. Since any of these differences may have contributed to differences in the results, we decided to begin our investigation by adopting all of the methods from B&S, in an attempt to replicate their original findings.

### Methods

#### Participants

Twenty-six students (25 female, mean age = 20 years, range between 18 and 22) from the University of Maryland, College Park participated in this experiment. All participants were native speakers of English and had normal or corrected-to-normal vision. All participants gave informed consent and received course credit for their participation. Data from two additional participants were excluded: one because accuracy on comprehension questions was too low (71%); the other because too many experimental items (25%) contained RTs greater than 2000 ms.

#### Design and materials

This experiment had a 2 × 2 within-participant design in which *main clause match* and *embedded match* were fully crossed. We used the original 24 sets of sentences in B&S's Experiment 1. These materials contained an unambiguously gendered proper name in both the main clause and embedded subject positions and therefore resembled the materials used in the proper name condition in Experiment 1 (see Table 3 for an example). In the original study half of the sentences used the feminine object pronoun ‘her,’ but sentences with feminine and masculine object pronoun were analyzed together. In order to increase the statistical power for examining the effects of the gender of the pronoun, we created 24 additional sets of sentences modeled after B&S's items, half of which used the feminine object pronoun. A complete set of experimental stimuli are available in the Supplementary Materials.

**Table 3:** **Experimental conditions and sample materials in Experiment 2**.

	**Embedded match**	**Embedded mismatch**
**MASCULINE OBJECT PRONOUN ‘HIM’**		
Main clause match (grammatical)	Arthur believed that Ben owed him a second chance to solve the problem.	Arthur believed that Meg owed him a second chance to solve the problem.
Main clause mismatch (ungrammatical)	Sheila believed that Ben owed him a second chance to solve the problem.	Sheila believed that Meg owed him a second chance to solve the problem.
**FEMININE OBJECT PRONOUN ‘HER’**		
Main clause match (grammatical)	Sheila believed that Meg owed her a second chance to solve the problem.	Sheila believed that Ben owed her a second chance to solve the problem.
Main clause mismatch (ungrammatical)	Arthur believed that Meg owed her a second chance to solve the problem.	Arthur believed that Ben owed her a second chance to solve the problem.

The 48 item sets were divided into four presentation lists, such that each list contained exactly one version of each item and 6 items in each condition. Each list also contained 100 filler sentences, which varied in length and syntactic complexity and contained other referential expressions (e.g., proper names and gender-neutral nouns) and anaphors (e.g., feminine pronouns and reflexives). Following B&S, a single word probe was selected for each experimental and filler item set. For half of the items, the probe word was selected from among the content words of the sentence—never the pronoun or either of the proper names. The location of the probe in the sentence (initial, medial, or final) was counterbalanced across items. For the other half of the items, words that did not occur in the sentence(s) were selected. Among these “no” probes, one third were semantic associates to a content word in the sentence (e.g., beach—ocean), one third were morphologically related to a word in the sentence (e.g., accepted—acceptance), and one third were neither semantically nor morphologically related to any content words in the sentence. Following B&S, comprehension questions were presented on one quarter of the trials. As in Experiment 1, responses to comprehension questions never required successful pronoun resolution. Finally, five additional complete trials were constructed to serve as practice trials.

#### Procedure

The procedure was similar to that of Experiment 1 with three critical differences. First, words were presented at the center of the screen. Second, at the end of each sentence, a probe word appeared at the center of the screen and the participant used the keyboard to indicate whether that probe word had occurred in the sentence. Finally, auditory feedback was provided to indicate accuracy on both of the secondary tasks. Testing sessions lasted approximately 30 min.

#### Analysis

As in Experiment 1, trials containing RTs greater than 2000 ms were excluded from the analysis. This affected 2.2% of the data. Initial statistical analyses were performed on data from all items, collapsing across pronoun gender. Data for each of the regions of interest were entered into a 2 × 2 repeated measures ANOVA with main clause match and embedded match as within-participant factors. We conducted two follow-up analyses to further examine potential differences between the present results and B&S's findings. We performed the same 2 × 2 repeated measures ANOVA on the subset of items taken from B&S to determine whether those items would show a different pattern. Finally, to examine the role of the pronoun gender, we added Gender as an additional factor to analyze all the items together. Data for each of the regions of interest were entered into a 2 × 2 × 2 repeated measures ANOVA with main clause match, embedded match and pronoun gender as within- participant factors.

### Results

Participants answered the comprehension questions and performed the probe recognition task with an average of 86.2 and 93.9% accuracy respectively.

#### All items

**Figure 2:**
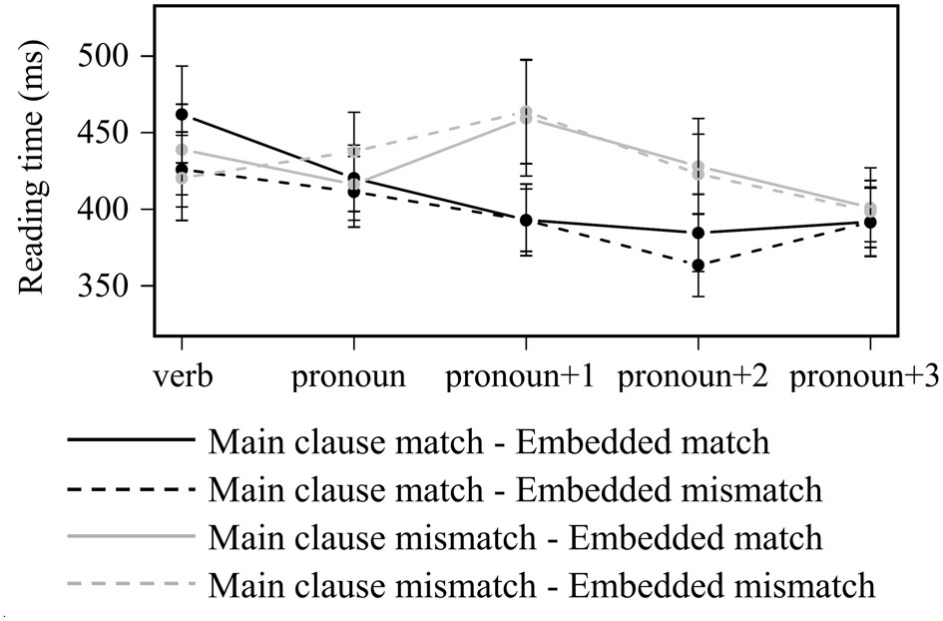
**Word-by-word reading times for all items in Experiment 2**. Error bars indicate standard error of the participant mean.

Main clause mismatch led to longer reading times across several regions (see Figure 2). The main effect of *main clause match* was significant in the by-items analysis in the *pronoun* region [422 ms vs. 400 ms; *F*_1(1, 25)_ = 3.57, *p* = 0.07; *F*_2(1, 47)_ = 4.87, *p* < 0.05], and in both analyses in the *pronoun+1* [447 ms vs. 392 ms; *F*_1(1, 25)_ = 12.0, *p* < 0.01; *F*_2(1, 47)_ = 16.9, *p* < 0.001] and *pronoun+2* [414 ms vs. 377 ms; *F*_1(1, 25)_ = 8.82, *p* < 0.01; *F*_2(1, 47)_ = 16.8, *p* < 0.001] regions. No other comparisons revealed a statistically significant difference (*p*'s > 0.05).

#### Badecker and Straub's (2002) items

A similar pattern of results was observed in a separate analysis of just the subset of items from B&S. Main clause mismatch led to longer reading times at the pronoun and the three subsequent regions. this main effect of *main clause match* was statistically significant in the *pronoun* region [418 ms vs. 386 ms; *F*_1(1, 25)_ = 5.26, *p* < 0.05; *F*_2(1, 23)_ = 7.25, *p* < 0.05], and in the by-items analysis in the *pronoun+1* region [438 ms vs. 392 ms; *F*_1(1, 25)_ = 3.89, *p* = 0.06; *F*_2(1, 23)_ = 4.81, *p* < 0.05]. In the *pronoun+2* region, there was an interaction between *main clause match* and *embedded match* which was significant in the by-items analysis [*F*_1(1, 25)_ = 3.57, *p* = 0.07; *F*_2(1, 23)_ = 5.72, *p* < 0.05]. This interaction had the pattern of an ungrammatical match effect: when the main clause subject mismatched the pronoun, RTs were longer when the embedded subject matched (431 ms vs. 383 ms). No other comparisons revealed a statistically significant difference (*p*'s > 0.05).

#### Pronoun gender

In a follow-up analysis which included pronoun gender as an additional factor, we continued to observe a main effect of *main clause match* across the *pronoun* [*F*_1(1, 25)_ = 3.63, *p* = 0.07; *F*_2(1, 47)_ = 6.18, *p* < 0.05], *pronoun+1* [*F*_1(1, 25)_ = 12.1, *p* < 0.01; *F*_2(1, 47)_ = 15.8, *p* < 0.001], and *pronoun+2* [*F*_1(1, 25)_ = 8.89, *p* < 0.01; *F*_2(1, 47)_ = 14.3, *p* < 0.001] regions. In addition, reading times were significantly longer for ‘him’ than for ‘her’ in the *pronoun* [426 ms vs. 396 ms, *F*_1(1, 25)_ = 12.0, *p* < 0.01; *F*_2(1, 47)_ = 9.46, *p* < 0.01] and *pronoun+1* [430 ms vs. 408 ms, *F*_1(1, 25)_ = 7.70, *p* < 0.05; *F*_2(1, 47)_ = 3.27, *p* = 0.08] regions. No other comparisons revealed a statistically significant difference (*p*'s > 0.05).

### Discussion

In this experiment we adopted the experimental materials and procedures used in B&S's Experiment 1 in an attempt to replicate their observation of a multiple match effect. This attempt was unsuccessful, as we once again failed to observe any sensitivity to the presence of multiple feature-matching candidate antecedents. Instead we replicated the key findings from our Experiment 1: a robust effect of grammaticality—longer reading times when the main clause subject mismatched the pronoun—and no facilitative interference effect or multiple match effect. When we looked at the subset of items taken from B&S's original study, we observed only a late ungrammatical match effect, similar to that observed in the modified common noun condition in Experiment 1. Across all cases, reading times were never modulated by the presence/absence of a feature-matching embedded subject when the main clause subject matched the pronoun in features.

## Experiments 3–5

Here we present three further attempts to explore the potential cause of comprehenders' sensitivity (or lack thereof) to the presence of multiple candidate antecedents when processing a pronoun. The design of these experiments was different from that of Experiments 1 and 2. To focus on potential multiple match effects, we removed the manipulation of the main clause subject—it always matched the pronoun in gender. We added a new manipulation of the *pronoun type*: object (‘him’) vs. possessive (‘his’). In the possessive condition, both the main clause and embedded subjects are structurally acceptable, so the pronoun is referentially ambiguous. Thus, if the multiple match effect is possible, we should certainly expect to see it in the possessive condition.

We also added a manipulation of the referential status of the embedded subject: referential (e.g., ‘the consultant’) vs. quantified (e.g., ‘every consultant’). This manipulation was originally motivated by the hypothesis that the multiple match effects observed by B&S could be related to the fact that local antecedents are acceptable in certain pragmatic contexts (Evans, 1980). Such effects might not be expected for quantified NPs (Reinhart, 1983). However, since we never observed a multiple match effect in any of our experiments, a full explanation of the theoretical motivation for this manipulation is beyond the scope of this paper. Here it serves only as a further test of the more basic questions about the structure sensitivity of the initial antecedent retrieval process across a wider range of sentences.

In Experiments 3 and 4 we used a moving-window self-paced reading paradigm. In Experiment 5 we used eye-tracking to examine comprehenders' eye movements while reading. In self-paced reading paradigms, reading must proceed in one direction, while in eye-tracking paradigms participants are free to skip or re-read parts of the sentence that they have previously read (or skipped). Thus, eye-tracking may be able to detect differences that only emerge in more naturalistic reading.

To preview, we never observed a multiple match effect in any of these experiments. In fact, comprehenders did not show increased reading times to a pronoun and its subsequent words, even in cases of genuine referential ambiguity, where multiple structurally acceptable and feature-matching candidate antecedents were available (in the possessive condition). Since the same design was used in all three experiments and they yielded minimally different results, below we report the methods and results of the three experiments together.

### Design and materials

The same experimental design was used across Experiments 3–5. We manipulated *pronoun type* (object vs. possessive pronoun), the embedded subject's *referential status* (referential vs. quantified) and the *embedded subject gender match* (match vs. mismatch) in a 2 × 2 × 2 within-participant design. A sample item set from Experiment 3 is shown in Table 4. The *pronoun type* determined the structural acceptability of the embedded subject as an antecedent for the pronoun: in the object pronoun condition (‘him’), only the main clause subject is structurally acceptable as an antecedent, while in the possessive pronoun condition (‘his’), both subjects are structurally acceptable. We only used singular masculine pronouns, as in Experiment 1, to avoid the lexical ambiguity of ‘her.’ The embedded subject, a stereotypically gender-biased common noun, was either quantified (e.g., ‘every consultant’) or referential (e.g., ‘the consultant’). The main clause subject always matched the pronoun in gender, but the embedded subject was manipulated to either match or mismatch the gender of the pronoun.

**Table 4:** **Experimental conditions and sample materials for Experiment 3**.

	**Embedded match (multiple match)**	**Embedded mismatch (single match)**
Referential	The lawyer believed that the stock broker who reported the fraud had deceived him/his *boss* about the extent of the illegal activity.	The lawyer believed that the secretary who reported the fraud had deceived him/his *boss* about the extent of the illegal activity.
Quantified	The lawyer believed that every stock broker who reported the fraud had deceived him/his *boss* about the extent of the illegal activity.	The lawyer believed that every secretary who reported the fraud had deceived him/his *boss* about the extent of the illegal activity.

There were minimal differences in the experimental materials across the three experiments. First, the main clause subjects were stereotypically male common nouns in Experiment 3, and unambiguously male proper names in Experiments 4 and 5. Second, to make the sentences more felicitous in the quantified condition, the embedded subject was modified by a relative clause or prepositional phrase in Experiment 3. In Experiment 4, the embedded subject was not modified; instead the experimental sentence was preceded by a context sentence. Finally, in Experiments 4 and 5, we added longer words (e.g., adverbs) immediately after the pronoun to reduce the likelihood of floor effects on reading times in the critical regions. The differences between the materials are illustrated in (6) and (7).

(6) *A sample item from Experiment 3:*
  The lawyer believed that the stock broker who reported the fraud had deceived him about the extent of the illegal activity.
(7) *A sample item from Experiments 4 and 5:*
  There appeared to be widespread fraud in the management of the hedge fund. Brian believed that the stock broker had deceived him repeatedly about the extent of the illegal activity.

A total of 80 sets of experimental sentences were used in Experiment 3; 64 sets were adapted and used in Experiments 4 and 5. A complete set of experimental stimuli are available in the Supplementary Materials. Gender bias of the common nouns was determined in an offline rating study (see Experiment 1, Design and Materials). On a scale from 1 (most likely female) to 7 (most likely male), female-biased nouns had an average rating 2.5 in Experiment 3 and 2.4 in Experiments 4 and 5. All female-biased nouns had an average rating below 4 (more likely to be female). Male-biased nouns had an average rating of 5.2 in Experiment 3 and 5.3 in Experiments 4 and 5. Most of them (76 of 80 main clause subjects and 75 of 80 embedded subjects in Experiment 3; 61 of 64 in Experiments 4 and 5) had an average rating above 4 (more likely to be male). The median rating difference between the female-biased and male-biased nouns within the same item was 2.6 points in both sets of stimuli.

In each experiment, experimental sentences were divided into 8 lists, each containing exactly one version of each item and the same number of items in each condition. A total of 80, 64, and 104 filler sentences of comparable length and structural complexity were used in Experiments 3, 4, and 5 respectively. Filler sentences contained other referential expressions (e.g., proper names and gender-neutral nouns) and anaphors (e.g., feminine pronouns and reflexives). In Experiments 3 and 5 every experimental and filler sentence was followed by a yes/no comprehension question; in Experiment 4 a yes/no comprehension question appeared following approximately one third of the trials (22 of 64 experimental and filler sentences respectively). The comprehension questions never referred to the referential dependency between the pronoun and its antecedent(s). The order of experimental and filler sentences was randomized across participants.

### Experiment 3

#### Participants

Twenty-six students (15 female, mean age = 22 years) from the University of Maryland, College Park participated in this experiment. All gave informed consent and were paid $10 per hour for their participation. Data from two additional participants were excluded: one because accuracy on comprehension questions was too low (<70%); the other because too many experimental items (>20%) contained RTs greater than 2000 ms.

#### Procedure

The procedure was identical to that of Experiment 1. Testing sessions lasted approximately 45 min.

#### Analysis

Data for different pronoun types were analyzed separately. Data from each region of interest were entered into a 2 × 2 repeated measures ANOVA with *referential status* and *embedded match* as within-participant factors. As in Experiments 1 and 2, trials containing RTs greater than 2000 ms were excluded from the analysis. This affected 1.2% of the data.

#### Results

Participants answered the comprehension questions with an average of 86.7% accuracy.

Grand average reading times in each ROI across all conditions are presented in Table 5.

**Table 5:** **Grand average reading times in each ROI across all conditions in Experiment 3**.

	**Object pronoun (him)**			**Possessive pronoun (his)**		
	**Pronoun**	**Pronoun+1**	**Pronoun+2**	**Pronoun**	**Pronoun+1**	**Pronoun+2**
Quantified, multiple match	331 (13)	319 (10)	300 (9)	325 (10)	328 (14)	343 (15)
Quantified, single match	330 (12)	329 (12)	301 (11)	322 (11)	335 (12)	351 (14)
Referential, multiple match	332 (14)	320 (12)	315 (13)	330 (14)	324 (13)	327 (12)
Referential, single match	332 (12)	322 (11)	309 (12)	327 (12)	330 (13)	325 (11)

No significant differences were observed in the *pronoun* and the *pronoun+1* region in either object pronoun or possessive pronoun condition. In the *pronoun+2* region, there was a significant main effect of *referential status* in the object pronoun condition [*F*_1(1, 25)_ = 5.05, *p* < 0.05; *F*_2(1, 79)_ = 4.12, *p* < 0.05]: reading times were shorter when the embedded subject was quantified (300 ms) compared to when it was referential (312 ms). A reversed pattern was observed in the possessive pronoun condition [main effect of *referential status*: *F*_1(1, 25)_ = 4.11, *p* = 0.05; *F*_2(1, 79)_ = 6.16, *p* < 0.05]: reading times were longer when the embedded subject was quantified (347 ms) compared to when it was referential (326 ms). No other comparisons revealed a statistically significant difference (*p*'s > 0.1).

### Experiment 4

#### Participants

Thirty-eight students (30 female, mean age = 22 years) from the University of Maryland, College Park participated in this experiment. All gave informed consent and received course credit or $10 per hour for their participation. Data from one additional participant were excluded due to low accuracy on the comprehension questions (<70%).

#### Procedure

The procedure was identical to that of Experiments 1 and 3.

#### Analysis

The analysis method was identical to that of Experiment 3. Outlier rejection (RTs > 2000 ms) affected 2.5% of the data.

#### Results

Participants answered the comprehension questions with an average of 90.0% accuracy.

Grand average reading times in each ROI across all conditions are presented in Table 6.

**Table 6:** **Grand average reading times in each ROI across all conditions in Experiment 4**.

	**Object pronoun (him)**			**Possessive pronoun (his)**		
	**Pronoun**	**Pronoun+1**	**Pronoun+2**	**Pronoun**	**Pronoun+1**	**Pronoun+2**
Quantified, multiple match	380 (17)	373 (13)	371 (12)	374 (15)	424 (22)	385 (15)
Quantified, single match	383 (17)	385 (15)	383 (13)	357 (15)	406 (19)	408 (16)
Referential, multiple match	386 (18)	381 (15)	360 (11)	362 (16)	400 (19)	394 (14)
Referential, single match	406 (24)	370 (11)	375 (13)	367 (18)	404 (18)	382 (15)

No significant differences were observed in any of the regions of interest in either of the pronoun conditions (all *p*'s > 0.1).

### Experiment 5

#### Participants

Twenty-four students (13 female, mean age = 22 years) from the University of Maryland, College Park participated in this experiment. All gave informed consent and received course credit or $10 per hour for their participation. Data collected from five additional participants were excluded due to problems with calibration.

#### Procedure

Participants were tested individually in a quiet room in one session lasting 45–60 min. Eye movements were recorded using an EyeLink 1000 eye-tracker (SR Research, Toronto, Ontario, Canada) interfaced with a PC computer. Participants were seated with their chin and forehead stabilized by the eye-tracker apparatus, 32 inches from an LCD monitor which displayed the stimuli. At this distance, 4.6 characters were displayed per degree of visual arc. The eye-tracker has an angular resolution of 0.25–0.5°. Viewing was binocular, but only the right eye was recorded. The sampling rate for recordings was 1000 Hz. Stimulus presentation and interface with the eye-tracker was implemented with the EyeTrack software suite (University of Massachusetts, Amherst).

Sentences were presented in 12-point fixed-width Courier font in two lines. The line break was located after the first word occurring at least 100 characters from the beginning of the line. Depending on the length of the first sentence, the line break generally fell around the fourth or fifth word of the second sentence—for example, between ‘the’ and ‘consultant’ in the sample item above. This location for the line break ensured that the pronoun and its following word appeared near the center of the second line. A calibration procedure was performed before the experiment, and re-calibration was carried out between trials as needed. Before the experiment began, each participant was instructed to read for comprehension as naturally as possible. Each trial began with only a gray square on the left edge of the display. The participant triggered the appearance of the sentences by fixating on the square, and pressed a button when they had finished reading to end the display of the item and trigger the presentation of the comprehension question.

#### Analysis

The initial stage of data analysis was carried out using EyeDoctor (UMass Amherst, http://www.psych.umass.edu/eyelab/software/). Trials with major tracker losses were excluded from the analyses. This resulted in the exclusion of 2.3% of all trials. Each trial was visually inspected to correct for small vertical drifts. Fixations of less than 80 ms in duration and within one character of the previous or following fixation were incorporated into this neighboring fixation. All remaining fixations shorter than 80 ms were excluded. Following Rayner and Pollatsek (1989), we assume that readers do not extract much information during such short fixations. We also excluded fixations longer than 800 ms.

We analyzed three regions, which corresponded to (i) the *pronoun* region, which included the pronoun and its immediately preceding word (i.e., the embedded verb), (ii) the *pronoun+1* region, which included the word immediately following the pronoun, and (iii) the *pre-final* region, which consisted of all words between the *pronoun+1* region and the sentence-final word (exclusive). Spaces between regions were included in the following region. Regions are indicated by brackets in the sample in (8).

(8) The international firm was to hold a press conference in the coming week. Patrick said that the consultant had [prepared him][sufficiently][to make a statement at the] meeting.

Standard eye-tracking measures (Rayner, 1998) were calculated for each region. We report three eye-tracking measures that are representative of early and late measures. *First-pass time* is the sum of all fixation times starting with the first fixation inside a region until the first fixation outside the region (either to the left or right) provided that the reader has not fixated subsequent text. For regions consisting of a single word, first-pass time corresponds to gaze duration (Rayner and Duffy, 1986). *Regression-path time* (e.g., Brysbaert and Mitchell, 1996) is the sum of all fixation times starting with the first fixation inside the region until the first fixation to the right of the region, again provided that the reader has not fixated subsequent text. Finally, *total time* is the sum of all fixations in a region. For all reading time measures, the data for a particular region were excluded if the reading time measure for that region was zero.

As in Experiments 3 and 4, data for different pronoun types were analyzed separately. Data from each region of interest were entered into a 2 × 2 repeated measures ANOVA with *referential status* and *embedded match* as within-participant and within-item factors. Below we report F1 and F2 statistics for data in the object pronoun condition and only F1 statistics for data in the possessive pronoun condition due to missing data in a small set of items in one of the regions or measures.

#### Results

Participants answered the comprehension questions with an average of 91.0% accuracy.

Grand average first pass time, regression path time, and total reading times in each ROI across all conditions are presented in Table 7.

**Table 7:** **Grand average first pass time, regression path time, and total reading times in each ROI across all conditions in Experiment 5**.

	**Object pronoun (him)**			**Possessive pronoun (his)**		
**Measure**	**Pronoun**	**Pronoun+1**	**Pre-final**	**Pronoun**	**Pronoun+1**	**Pre-final**
**FIRST-PASS TIME**						
Quantified, multiple match	324 (23)	285 (12)	790 (50)	334 (22)	297 (18)	1034 (62)
Quantified, single match	338 (24)	290 (16)	882 (56)	302 (22)	276 (10)	1011 (72)
Referential, multiple match	309 (15)	275 (12)	827 (50)	324 (24)	290 (16)	1005 (56)
Referential, single match	289 (16)	310 (16)	841 (64)	328 (19)	270 (14)	1063 (68)
**REGRESSION PATH TIME**						
Quantified, multiple match	471 (36)	394 (24)	1365 (165)	473 (38)	405 (30)	1518 (103)
Quantified, single match	436 (36)	422 (39)	1486 (150)	519 (68)	443 (36)	1777 (149)
Referential, multiple match	477 (33)	406 (43)	1320 (142)	476 (33)	354 (24)	1668 (154)
Referential, single match	456 (33)	423 (27)	1492 (147)	475 (35)	342 (25)	1923 (168)
**TOTAL TIME**						
Quantified, multiple match	549 (45)	420 (35)	1205 (110)	545 (45)	402 (33)	1533 (122)
Quantified, single match	544 (42)	417 (34)	1268 (107)	566 (38)	409 (20)	1567 (121)
Referential, multiple match	552 (44)	411 (28)	1218 (107)	535 (48)	407 (31)	1472 (124)
Referential, single match	533 (38)	428 (29)	1226 (99)	569 (45)	359 (24)	1591 (139)

***Object pronoun condition (him)***. Repeated measures ANOVA revealed a significant main effect of *referential status* on first-pass time in the pronoun region [*F*_1(1, 23)_ = 4.81, *p* < 0.05; *F*_2(1, 63)_ = 3.41, *p* < 0.1]: reading times were longer when the embedded subject was quantified than when it was referential. No other comparisons revealed a statistically significant difference (*p's > 0.05*).

***Possessive pronoun condition (his)***. In the *pronoun+1* region, there was a significant main effect of *referential status* on regression path time [*F*_1(1, 23)_ = 7.77, *p* < 0.05]: reading times were longer when the embedded subject was quantified than when it was referential. This effect was reversed in the *pre-final* region, in which regression path time was significantly shorter in the quantified conditions than in the referential conditions [*F*_1(1, 23)_ = 6.27, *p* < 0.05]. No other comparisons revealed a statistically significant difference (*p*'s > 0.05).

### Discussion

In Experiments 3–5 we examined whether comprehenders are sensitive to the presence of multiple feature-matching candidate antecedents, in cases where both candidates are structurally acceptable (‘his’) and in cases where only one is (‘him’).

The results were largely the same across all three experiments, and consistent with the findings of Experiments 1 and 2. Comprehenders were not sensitive to the gender match of the embedded subject, regardless of its referential status. Surprisingly, this also held in the ‘his’ condition, where we expected to observe a multiple match effect due to the referential ambiguity. This suggests that resolving this referential ambiguity did not lead to any observable processing cost, or that comprehenders did not in fact resolve it online. We will return to discuss this in more detail in the General Discussion.

## General discussion

Our goal in this paper was to investigate the role of structural constraints in the early stages of pronoun resolution—specifically, the initial retrieval of potential antecedents. We considered two hypotheses. Under the Simultaneous Constraints hypothesis, the initial retrieval would return a set of candidate antecedents constrained by both structural and agreement criteria. Under the Agreement First hypothesis, the initial retrieval would be constrained only by agreement features, while structural constraints would come into play later. Across all our experiments, the results supported the Simultaneous Constraint hypothesis.

In Experiments 1 and 2, we found that comprehenders are sensitive to structural constraints on antecedents as early as agreement constraints. Across all five experiments, we never observed any facilitative interference from the structurally unacceptable potential antecedent. Evidence for inhibitory interference was sparse: there were no instances of multiple match effects, and only one instance of an ungrammatical match effect, which emerged later than the initial sensitivity to structural constraints. Thus we have strong, consistent evidence for structure sensitivity in the earliest stages of pronoun resolution.

### No facilitative interference

The consistent lack of facilitative interference effects speaks against the Agreement First hypothesis. If the initial stages of pronoun resolution used only agreement features to identify a set of candidate antecedents, then reading times immediately following a pronoun should be sensitive only to the features of potential antecedents, not their structural position. The presence of a feature-matching (albeit structurally unacceptable) candidate would facilitate processing in sentences with no grammatical antecedent. We never observed such a pattern: reading times were always longer when the main clause subject mismatched the pronoun in gender, regardless of whether the embedded subject matched the pronoun in gender.

Some researchers have argued that studies may fail to observe interference effects due to a lack of power. If the predicted pattern of facilitative interference occurred with extremely small effect sizes, we could have failed to detect it even with multiple studies. We think this is unlikely, based on comparison with a case where facilitative interference is observed readily, without large numbers of participants and items: subject-verb agreement (production: Bock and Miller, 1991; comprehension: Staub, 2009, 2010; Wagers et al., 2009). For example, in ungrammatical sentences like (9), reading times on the verb ‘praise’ are shorter when a plural NP (‘the musicians’) is present in the context (as in 9b), compared to when only singular NPs are present (as in 9a).

(9) a. ^*^The musician who the reviewer praise so highly will probably win a Grammy.
  b. ^*^The musicians who the reviewer praise so highly will probably win a Grammy.

There is good evidence that facilitative interference in subject-verb agreement arises because the retrieval of the subject triggered by the verb is guided primarily by agreement features, not structure (Wagers et al., 2009). If so, under the Agreement First hypothesis, we would expect facilitative interference effects for subject-verb agreement and pronoun resolution to look the same, all things being equal. Of course, all things are not equal. However, the sentences we tested do favor the possibility of facilitative interference: the structurally unacceptable potential antecedent (the embedded subject) is closer to the pronoun both linearly and structurally, so it should be more highly activated in memory than the structurally acceptable potential antecedent at the point when the pronoun triggers the retrieval process. Thus, if antecedent retrieval for pronouns were unconstrained by structure, like subject retrieval for verb agreement, we would expect effect sizes at least as large as those observed in studies of subject-verb agreement, which should therefore be observable in experiments with the same power.

The lack of facilitative interference effects aligns pronouns with reflexives, which also resist interference from structurally unacceptable potential antecedents. Thus, there seems to be a broad division between the processing of agreement dependencies, which show the hallmarks of Agreement First retrieval, and the processing of referential dependencies like pronouns and reflexive (Dillon et al., 2013; but see Parker et al., 2012). Future research will need to establish ways in which the processing of referential and agreement dependencies differ (or not). This will likely provide insights into how different kinds of linguistic information are represented and accessed in memory.

### No multiple match or referential ambiguity effects

**Figure 3:**
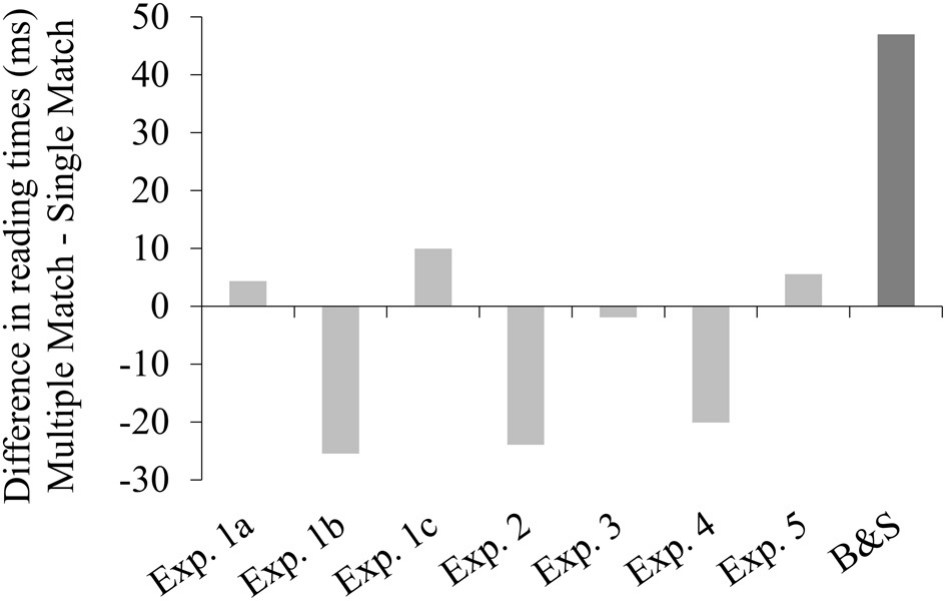
**Difference in reading times between the multiple match (main clause match/embedded match) and single match (main clause match/embedded mismatch) conditions in the *pronoun+1* region across Experiments 1–5, compared to Badecker and Straub's (2002) Experiment 1**.

Another important finding of the current study is that we never observed the multiple match effect reported by Badecker and Straub (2002). In this case, we need not worry about a lack of power to detect the effect: all of our experiments had more participants and items than Badecker and Straub's, resulting in 1.5–5 times as many relevant data points in each experiment. Figure 3 compares the lack of multiple match effect in the *pronoun+1* region across our five experiments to the rather sizeable effect observed in Badecker and Straub's Experiment 1.

In fact, the multiple match effect seems to be quite rare in the literature. Several other studies include the relevant comparison (Clifton et al., 1999, Experiment 3; Lee and Williams, 2008, Experiments 1 and 2; Patterson et al., 2014), but the effect has only been reported in one (Nicol, 1997; cited in Nicol and Swinney, 2003). In that study, the effect was driven by trials where the participant failed to identify the correct referent for the pronoun in a comprehension question. Nicol and Swinney (2003) therefore suggest that the presence of a multiple match effect depends on the participants' mode of reading.

We note, however, that the availability of more than one potential antecedent—even when they are all grammatically acceptable—does not necessarily lead to increased processing costs. In the possessive conditions of Experiments 3–5, the possessive pronoun ‘his’ was referentially ambiguous when it matched both the main clause and embedded subjects in features (e.g., ‘The executive insisted that the consultant who worked on the project should prepare his client for the weekly press meeting’). This referential ambiguity was not associated with any observable processing cost: embedded subject match never impacted comprehenders' reading time profiles in the possessive pronoun condition.

Although the lack of cost for ambiguity may seem surprising, such effects are often absent in studies comparing ambiguous and unambiguous pronouns in reading comprehension (e.g., Caramazza et al., 1977; Lee and Williams, 2008; Cunnings and Sturt, 2012; cf. MacDonald and MacWhinney, 1990; Garnham et al., 1995; Arnold et al., 2000; Nieuwland et al., 2007). The pronoun may be resolved using discourse constraints or heuristic strategies (e.g., first-mention bias: Corbett and Chang, 1983; implicit verb causality: Caramazza et al., 1977). Further, effects of referential ambiguity can also be modulated by other factors such as individual differences in working memory span (Nieuwland and Van Berkum, 2006), depth of processing (Stewart et al., 2007) and task demands (Yee and Heller, 2012). These factors might encompass the “mode of reading” idea suggested by Nicol and Swinney (2003).

Thus, even though both the main clause and embedded subjects were plausible antecedents for the possessive pronoun in the present experimental materials, various factors may have contributed to the lack of an ambiguity effect. Future work will be needed to determine how task and individual differences may explain the variation across studies. What is clear is that multiple match effects are far from being the dominant pattern in cases of multiple feature-matching intrasentential antecedents.

### Limited ungrammatical match effects

We observed the “ungrammatical match” type of inhibitory interference in two cases: the *modified common noun* condition of Experiment 1, and the items in Experiment 2 drawn from Badecker and Straub's (2002) study. In these cases, the presence of a feature-matching but structurally unacceptable potential antecedent led to longer reading times when no grammatical antecedent was available.

Following Sturt's (2003) proposal, we suggest that initial failure to retrieve an acceptable antecedent for a reflexive or pronoun may trigger reanalysis processes leading to increased processing time when a structurally unacceptable potential antecedent matches the pronoun in features. Specifically, to recover an antecedent for the pronoun or reflexive, a feature-matching antecedent in a structurally unacceptable position may be considered. This consideration leads to increased processing time compared to the case when there are no feature-matching candidates at all to be considered. This account makes two predictions. First, sensitivity to a structurally unacceptable potential antecedent should be present only when no grammatical antecedents are available. Second, the effect should be delayed relative to the effect of grammaticality. Both of these predictions are compatible with the evidence available thus far. For instance, while Sturt (2003) observed an effect of grammaticality in first pass reading times, inhibitory interference in ungrammatical sentences was present only in second pass reading times. Correspondingly, in our Experiment 1, while there was an effect of grammaticality in the *pronoun+1* region, the inhibitory interference in ungrammatical sentences was observed only in the *pronoun+2* region.

Note, however, that this effect has only been observed in a subset of the existing studies that allowed the relevant comparison. It emerged in the modified common noun condition of our Experiment 1, but neither of the other embedded subject types, and in only half the items in Experiment 2. Other studies with similar designs have also failed to find any inhibitory interference in ungrammatical sentences (e.g., Clifton et al., 1999; Badecker and Straub, 2002; Lee and Williams, 2008). We take the inconsistency of the effect to suggest that initial failures to retrieve a structurally acceptable and feature-matching antecedent do not always trigger additional reanalysis processes, even when a feature-matching and structurally unacceptable potential antecedent is available. Future research will be needed to explore whether and how this effect may be modulated by factors such as task demands and the memory representation of the potential antecedents.

## Conclusion

In the current study we examined whether structural constraints (Binding Principle B) impact the initial memory retrieval process alongside agreement constraints during pronoun interpretation. We argue that both the current results and previous evidence support the hypothesis that agreement features and structural constraints are used simultaneously in the process of pronoun interpretation.

### Conflict of interest statement

The authors declare that the research was conducted in the absence of any commercial or financial relationships that could be construed as a potential conflict of interest.
